# Genomic analyses of bacterial porin-cytochrome gene clusters

**DOI:** 10.3389/fmicb.2014.00657

**Published:** 2014-11-26

**Authors:** Liang Shi, James K. Fredrickson, John M. Zachara

**Affiliations:** Pacific Northwest National LaboratoryRichland, WA, USA

**Keywords:** extracellular electron transfer, outer membrane, c-type cytochromes with multiple hemes, porin-cytochrome protein complex, metal reduction

## Abstract

The porin-cytochrome (Pcc) protein complex is responsible for trans-outer membrane electron transfer during extracellular reduction of Fe(III) by the dissimilatory metal-reducing bacterium *Geobacter sulfurreducens* PCA. The identified and characterized Pcc complex of *G. sulfurreducens* PCA consists of a porin-like outer-membrane protein, a periplasmic 8-heme *c*-type cytochrome (*c*-Cyt) and an outer-membrane 12-heme *c*-Cyt, and the genes encoding the Pcc proteins are clustered in the same regions of genome (i.e., the *pcc* gene clusters) of *G. sulfurreducens* PCA. A survey of additionally microbial genomes has identified the *pcc* gene clusters in all sequenced *Geobacter* spp. and other bacteria from six different phyla, including *Anaeromyxobacter dehalogenans* 2CP-1, *A. dehalogenans* 2CP-C, *Anaeromyxobacter* sp. K, *Candidatus* Kuenenia stuttgartiensis, *Denitrovibrio acetiphilus* DSM 12809, *Desulfurispirillum indicum* S5, *Desulfurivibrio alkaliphilus* AHT2, *Desulfurobacterium thermolithotrophum* DSM 11699, *Desulfuromonas acetoxidans* DSM 684, *Ignavibacterium album* JCM 16511, and *Thermovibrio ammonificans* HB-1. The numbers of genes in the *pcc* gene clusters vary, ranging from two to nine. Similar to the metal-reducing (Mtr) gene clusters of other Fe(III)-reducing bacteria, such as *Shewanella* spp., additional genes that encode putative *c*-Cyts with predicted cellular localizations at the cytoplasmic membrane, periplasm and outer membrane often associate with the *pcc* gene clusters. This suggests that the Pcc-associated *c*-Cyts may be part of the pathways for extracellular electron transfer reactions. The presence of *pcc* gene clusters in the microorganisms that do not reduce solid-phase Fe(III) and Mn(IV) oxides, such as *D. alkaliphilus* AHT2 and *I. album* JCM 16511, also suggests that some of the *pcc* gene clusters may be involved in extracellular electron transfer reactions with the substrates other than Fe(III) and Mn(IV) oxides.

## Introduction

*Geobacter* spp. are a group of Gram-negative bacteria whose hallmark feature is transfer of metabolically-derived electrons to appropriate electron-accepting substrates external to the bacterial cells, such as oxidized metals, electrodes and even other microorganisms (Lovley et al., 2004, 2011; Summers et al., 2010). *Geobacter* spp. are found in a wide range of habitats and are distributed world-wide. They are important in different environmental processes, including biogeochemical cycling of carbon and iron and attenuation of metal, radionuclide, and organic contaminants. *Geobacter* spp. have also been harnessed for a variety of biotechnology applications, such as bioremediation of contaminants in the subsurface sediments, generation of electrical current as microbial fuel cells, and electrosynthesis of organic compounds (Lovley et al., 2004, 2011).

In order to use extracellular substrates as terminal electron acceptors, *Geobacter* spp. have developed pathways to transfer electrons from the quinone/quinol pool in the cytoplasmic membrane, across the periplasm and the outer membrane to the extracellular substrates (Lovley, 2006; Weber et al., 2006; Shi et al., 2007, 2009; Bird et al., 2011). Previously, we identified and characterized a trans-outer membrane porin-cytochrome (Pcc) protein complex for transferring electrons across the outer membrane during extracellular reduction of Fe(III) by *G. sulfurreducens* PCA. The identified Pcc complex of *G. sulfurreducens* PCA consists of a porin-like outer-membrane protein (OmbB or OmbC), a periplasmic 8-heme *c*-type cytochrome (*c*-Cyt, OmaB or OmaC) and an outer-membrane 12-heme *c*-Cyt (OmcB or OmcC). The genes that encode Pcc proteins are adjacent to each other in the genome (i.e., the *pcc* gene cluster) of *G. sulfurreducens* PCA that possesses total four *pcc* gene clusters, two of which are involved in extracellular reduction of Fe(III)-citrate and ferrihydrite [a poorly crystalline Fe(III) oxide]. Isolated Pcc complex reconstituted in proteoliposomes transfers electrons from the reduced methyl viologen inside the liposomes across the lipid-bilayer to Fe(III)-citrate or ferrihydrite. The *pcc* gene clusters are present in all eight sequenced *Geobacter* genomes and 11 other phylogenetically diverse bacterial genomes. Widespread distribution of the *pcc* gene clusters in phylogenetically diverse bacteria reflects the importance of Pcc proteins in trans-outer membrane electron transfer by the Gram-negative bacteria (Liu et al., 2014).

Furthermore, the characterized function and organization of the Pcc complex of *G. sulfurreducens* PCA are very similar to that of the Mtr (i.e., metal-reducing) porin-cytochrome extracellular electron transfer complex in *Shewanella oneidensis* MR-1, despite the fact that Pcc and Mtr proteins are phylogenetically unrelated (Liu et al., 2014). In *S. oneidensis* MR-1, the characterized Mtr porin-cytochrome protein complex also consists of a porin-like outer-membrane protein (MtrB), a periplasmic 10-heme *c*-Cyt (MtrA) and an outer-membrane 10-heme *c*-Cyt (MtrC), and is responsible for electron transfer across the outer membrane during extracellular reduction of Fe(III) oxides (Hartshorne et al., 2009; Richardson et al., 2012; White et al., 2013). The Pcc and Mtr complexes appear to have evolved independently to a common functional role in mediating electron transfer across the bacterial outer membrane. The observed functional and organizational similarity between the Pcc and Mtr protein complexes collectively demonstrates that porin-cytochrome protein complex is a common mechanism shared by different groups of Gram-negative bacteria for trans-outer membrane electron transfers (Liu et al., 2014).

Despite detailed characterization of the Pcc complexes of *G. sulfurreducens* PCA and discovery of widespread distribution of *pcc* gene clusters in the Gram-negative bacteria, other features of the *pcc* gene clusters, such as their genetic organization, phylogenetic relationship and potential biological functions, had not been previously investigated. In this report, we further analyzed the characteristics of identified bacterial *pcc* gene clusters.

## Approach

The *pcc* gene clusters were identified as previously described (Liu et al., 2014). Briefly, the amino acid sequences of OmaB/OmaC, OmbB/OmbC, OmcB, and OmcC were used as the templates to search for the open reading frames (ORFs) whose predicted polypeptide products displayed similarity to OmaB/OmaC, OmbB/OmbC, OmcB, and OmcC by BLAST (*E* < 0.01) (Altschul et al., 1990; Shi et al., 2012b). The microbial genomes that were public available in November 4th, 2013 were searched. To confirm that any tentatively identified ORFs indeed possessed the trans-outer membrane motifs and/or the heme-binding motifs (CX_2_CH), its DNA-derived amino acid sequence was analyzed by Hidden Markov Model method for the porin-like outer-membrane protein and/or visually inspected for the motif CX_2_CH (Bagos et al., 2004a,b; Shi et al., 2012b). After confirmation, the DNA-derived amino acid sequences of *pcc* and their associated genes were used for phylogenetic analyses and BLAST search (*E* < 0.01) (Altschul et al., 1990). The phylogenetic analyses were performed with MEGA6 and confidence levels were determined by analyzing 1000 bootstrap replications (Tamura et al., 2013). The lipoproteins and cytoplasmic membrane proteins were predicted with previously described methods (Krogh et al., 2001; Juncker et al., 2003).

## Results and discussion

### Overview

**Figure 1:**
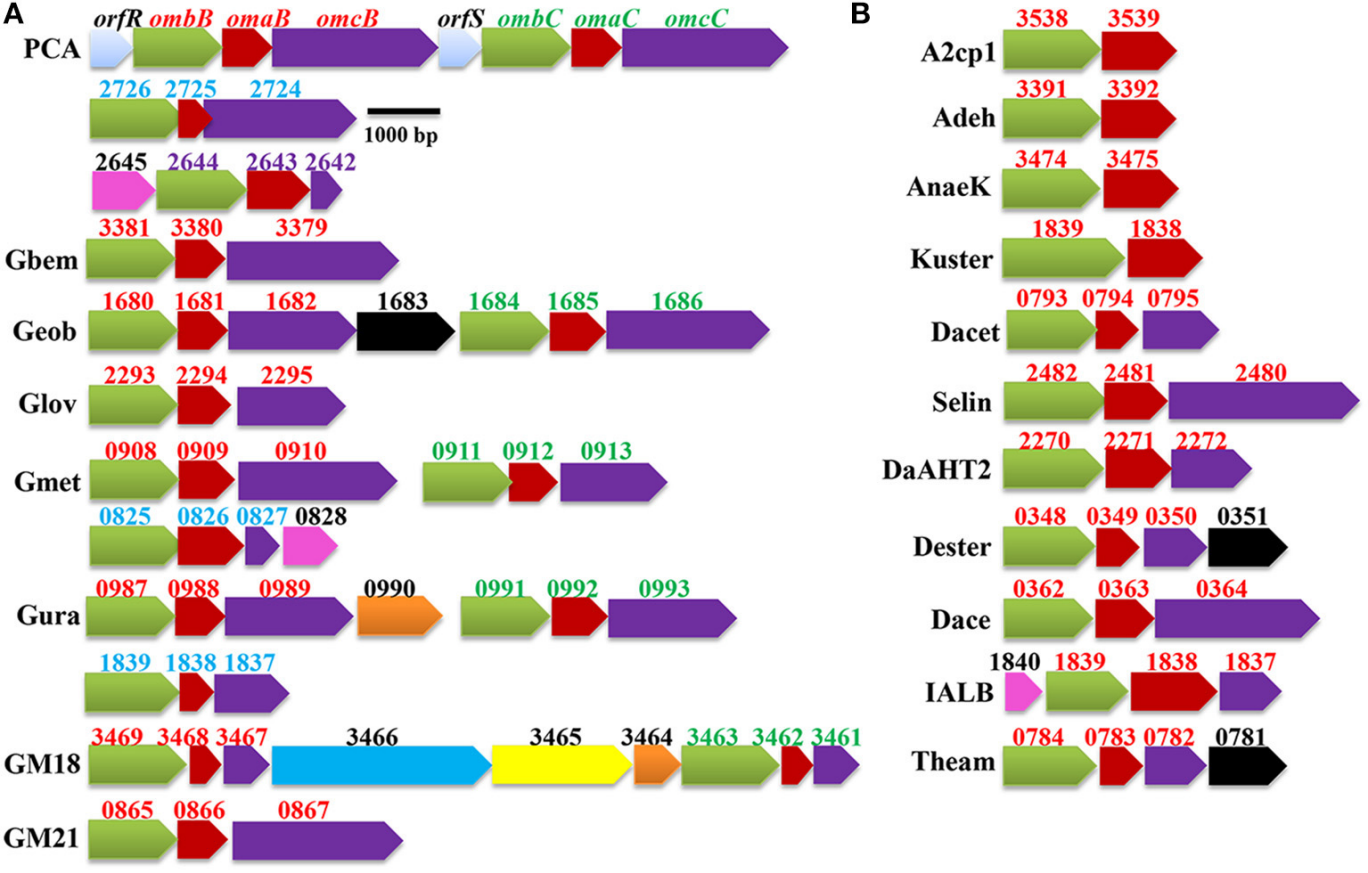
**Identified *pcc* gene cluster in *Geobacter* spp. (A) and other bacteria (B)**. The genes encoding different type of proteins are labeled with different colors: green/the Pcc porin-like outer-membrane proteins; red/the Pcc periplasmic *c*-type cytochromes (*c*-Cyts); purple/the Pcc outer-membrane *c*-Cyts; black/the cytoplasmic membrane *c*-Cyts; pink/the periplasmic *c*-Cyts; yellow/the porin-like outer-membrane *c*-Cyt; light blue/transcriptional factors; dark blue/chitinase and orange/hypothetic proteins. The numbers displayed above the gene clusters are part of their locus tags whose letter parts are displayed at left side of the gene clusters with exception that PCA is displayed for the gene clusters identified from *G*. *sulfurreducens* PCA. The numbers for the *pcc* genes are in red, green, blue or purple, while the numbers for the gene associated with *pcc* genes are in black. Gbem: *Geobacter bemidjiensis* Bem; Geob*: Geobacter* sp. FRC-32; Glov: *Geobacter lovleyi* SZ; Gmet: *Geobacter metallireducens* GS-15; Gura: *Geobacter uraniireducens* Rf4; GM18: *Geobacter* sp. M18; GM21: *Geobacter* sp. M21; A2cp1: *Anaeromyxobacter dehalogenans* 2CP-1; Adeh: *A. dehalogenans* 2CP-C; AnaeK: *Anaeromyxobacter* sp. K; Kuster: *Candidatus* Kuenenia stuttgartiensis; Dacet: *Denitrovibrio acetiphilus* DSM 12809; Selin: *Desulfurispirillum indicum* S5; DaAHT2: *Desulfurivibrio alkaliphilus* AHT2; Dester: *Desulfurobacterium thermolithotrophum* DSM 11699; Dace: *Desulfuromonas acetoxidans* DSM 684; IALB: *Ignavibacterium album* JCM 16511; and Theam: *Thermovibrio ammonificans* HB-1.

**Figure 2:**
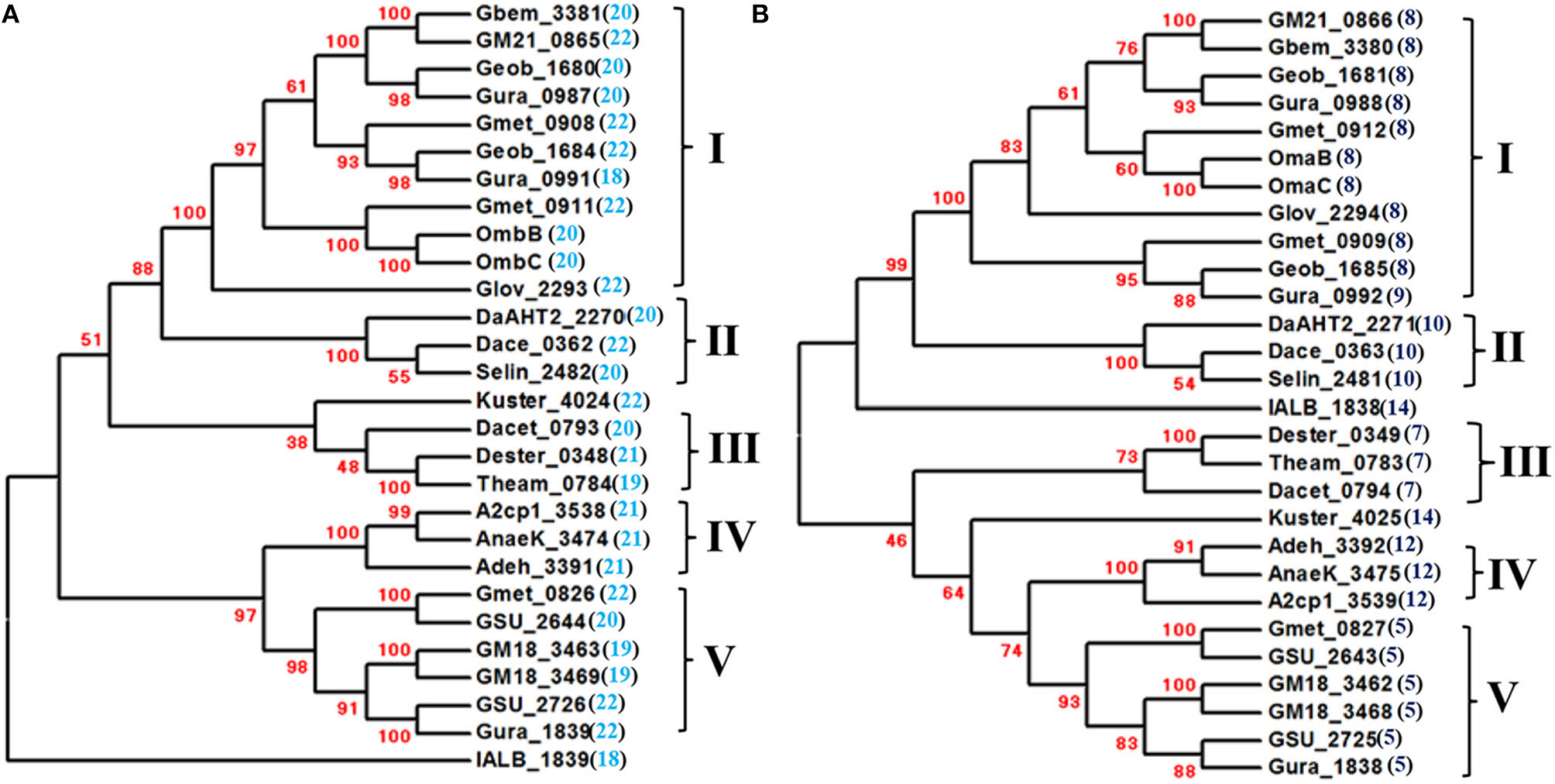
**Phylogenetic analyses of the Pcc porin-like outer-membrane proteins (A) and periplasmic *c*-type cytochromes (B)**. The phylogenetic trees were constructed with MEGA6 and confidence levels are indicated in the major nodes by the bootstrap values (%) in red. The numbers in the parenthesis next to the locus tags are the numbers of their predicted trans-outer membrane motifs of the porin-like outer-membrane proteins **(A)** and heme-binding motifs of the periplasmic *c*-type cytochromes **(B)**. The phylogenetic groups of the Pcc porin-like outer-membrane proteins **(A)** and periplasmic *c*-type cytochromes **(B)** are indicated by Roman numerals. The trees are not drawn to scale.

**Figure 3:**
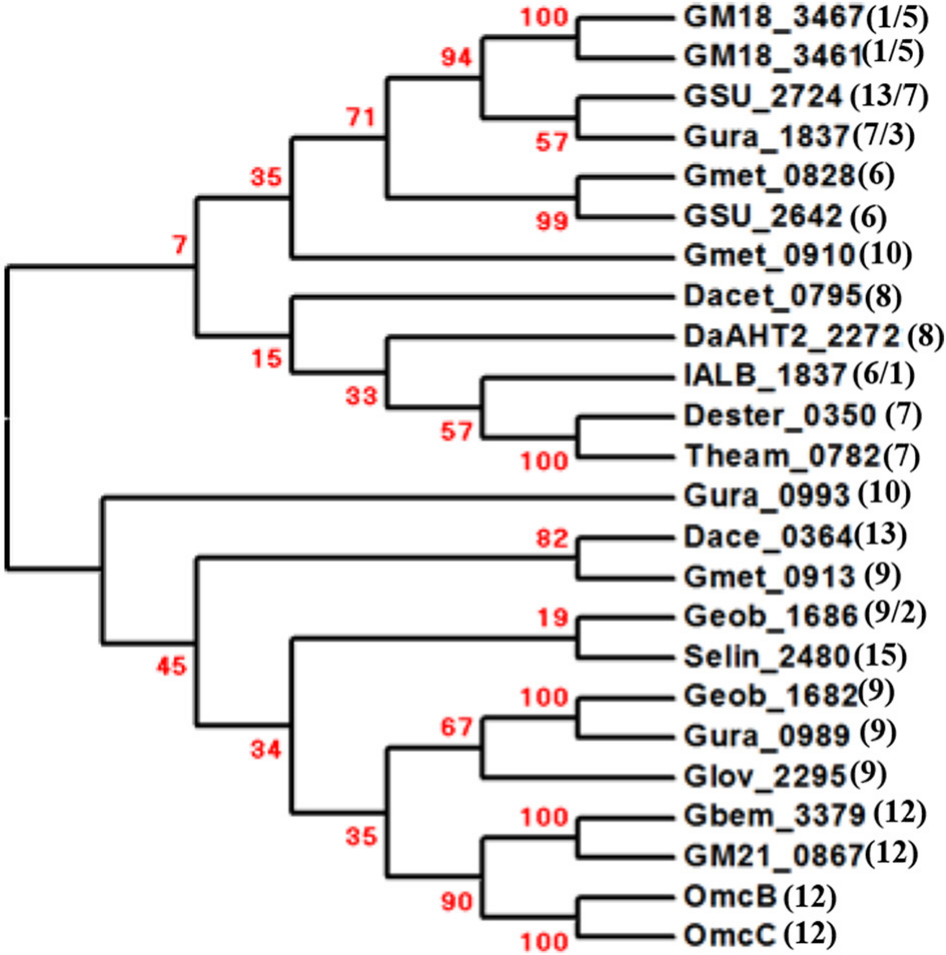
**Phylogenetic analyses of the Pcc outer-membrane *c*-type cytochromes**. The phylogenetic trees were constructed with MEGA6 and confidence levels are indicated in the major nodes by the bootstrap values (%) in red. The numbers in the parenthesis next to the locus tags are the numbers of their predicted typical (CX_2_CH)/atypical (CX_3-15_CH) heme-binding motifs of the *c*-type cytochromes. The tree is not drawn to scale.

As shown in Figure 1, the *pcc* gene clusters were found all sequenced *Geobacter* genomes. These include the genomes of *G. sulfurreducens* PCA, *G. bemidjiensis* Bem, *G. daltonii* FRC-32, *G. lovleyi* SZ, *G. metallireducens* GS-15, *G. uraniireducens* Rf4, *Geobacter* spp. M18, and *Geobacter* spp. M21. Numbers of the *pcc* gene clusters found in *Geobacter* genomes varied, ranging from one in *G. bemidjiensis* Bem and *Geobacter* sp. M21 to four in *G. sulfurreducens* PCA. Notably, for the *Geobacter* genomes with > one *pcc* gene clusters, at least two *pcc* gene clusters were consistently adjacent to each other. In *G. sulfurreducens* PCA, the *orfS-ombB-omaB-omcB*, and *orfR-ombC-omaC-omcC* gene clusters that are adjacent to each other are the result of gene duplication because at the amino acid sequence level, OmbB/OmbC and OmaB/OmaC are 100% identical, respectively, and OrfR/OrfS and OmcB/OmcC are 99 and 71% identical, respectively (Leang et al., 2003; Leang and Lovley, 2005; Aklujkar et al., 2013; Liu et al., 2014). Given that GM18_3461/GM18_3467, GM18_3462/GM18_3468, and GM18_3463/GM18_3469 are 100% identical at their amino acid sequence levels, respectively, the adjacent GM18_3461-GM18_3462-GM18_3463 and GM18_3467-GM18_3468-GM18_3469 gene clusters of *Geobacter* sp. M18 are also the result of gene duplication. The corresponding components of the *pcc* gene clusters that are adjacent in the genomes of *G. daltonii* FRC-32, *G. metallireducens* GS-15, and *G. uraniireducens* Rf4 are <64% identical at the amino acid sequence level, suggesting that they are unlikely to have arisen as a result of gene duplication (Figures 1A, 2, 3).

The *pcc* gene clusters were also found in the genomes of a group of phylogenetically and functionally diverse bacteria. These include the dissimilatory Fe(III)-reducing bacteria *Anaeromyxobacter dehalogenans* 2CP-1, *A. dehalogenans* 2CP-C, *Anaeromyxobacter* sp. K, and *Desulfuromonas acetoxidans* DSM 684; the selenate [SeO^2−^_4_]- and selenite [SeO^2−^_3_]-respiring bacterium *Desulfurispirillum indicum* S5; the elemental sulfur (S^0^)-reducing bacteria *Desulfurivibrio alkaliphilus* AHT2, *Desulfurobacterium thermolithotrophum* DSM 11699, and *Thermovibrio ammonificans* HB-1; the anammox bacterium *Candidatus* Kuenenia stuttgartiensis; the dissimilatory nitrate-reducing bacterium *Denitrovibrio acetiphilus* DSM 12809 and the moderately thermophilic chemoheterotrophic bacterium *Ignavibacterium album* JCM 16511 (Figure 1B) (Roden and Lovley, 1993; L'Haridon et al., 1998; Myhr and Torsvik, 2000; Narasingarao and Haggblom, 2007; Sorokin et al., 2008; Iino et al., 2010; Rauschenbach et al., 2011a; Giovannelli et al., 2012; Nissen et al., 2012; Speth et al., 2012). Only one *pcc* gene cluster was found in the genomes of each of these searched microorganisms (Figure 1B).

Notably, the *pcc* gene clusters identified from three *Anaeromyxobacter* spp. and *Candidatus* Kuenenia stuttgartiensis have only two genes that are predicted to encode a porin-like outer-membrane protein and a periplasmic *c*-Cyt with 12 or 14 heme-binding motifs, respectively, which is similar to some of the *mtr* gene clusters (Figures 1, 2A,B). The *mtr* genes found in other metal-reducing bacteria, such as *Shewanella* spp., are also clustered in the bacterial genomes (Fredrickson et al., 2008; Liu et al., 2012; Shi et al., 2012b). Some of the *mtr* gene clusters, especially those involved in Fe(II) oxidation, lack the genes encoding the outer membrane *c*-Cyts, such as *mtrC* gene (Jiao and Newman, 2007; Hartshorne et al., 2009; Liu et al., 2012; Shi et al., 2012b; Emerson et al., 2013). In *S. oneidensis* MR-1, the MtrAB or MtoA/MtrB complex alone that possess only 10 hemes can transfer electrons across the outer membrane for extracellular reduction of Fe(III) (Coursolle and Gralnick, 2010; Liu et al., 2012). Moreover, MtrAB can be co-purified without MtrC and the purified MtrAB complex reconstituted in proteoliposomes can transfer electrons across the lipid-bilayer of the liposomes (Hartshorne et al., 2009; White et al., 2013). The 10-heme MtrA polypeptide contains 333 amino acid residues with a calculated molecular mass of 36.0 kDa. Insights into the MtrA structure, determined by small-angle X-ray scattering and analytical ultracentrifugation, suggest that this protein is rod-shaped with length of 104 Å (Firer-Sherwood et al., 2011). The Pcc periplasmic *c*-Cyts of *Anaeromyxobacter* spp. and the *Candidatus* Kuenenia stuttgartiensis possess 338–344 amino acid residues with calculated molecular masses of 36.9–39.2 kDa and are predicted to have 12–14 hemes. If they are structurally similar to MtrA, the Pcc periplasmic *c*-Cyts of *Anaeromyxobacter* spp. and the *Candidatus* Kuenenia stuttgartiensis along with their respective porin-like outer-membrane proteins could potentially provide a span sufficient to transfer electrons across the outer membrane even without the outer-membrane *c*-Cyt counterparts.

In all cases, a gene encoding a porin-like outer-membrane protein is always associated with a gene encoding a periplasmic *c*-Cyt and in most cases a gene encoding an outer-membrane *c*-Cyt (Figures 1A,B).

### The *pcc*-associated genes

Most of the *mtr* gene clusters contain additional genes encoding *c*-Cyt (Fredrickson et al., 2008; Shi et al., 2011, 2012b; Liu et al., 2012, 2013). These Mtr-associated *c*-Cyts are involved in extracellular reduction of Fe(III) oxides on the exterior side of the outer membrane, such as OmcA and UndA; quinone/quinol redox cycling in the cytoplasmic membrane, such as CymA; and probably electron transfer in the periplasm, such as MtrK/MtoD (Lower et al., 2009; Reardon et al., 2010; Shi et al., 2011, 2012a,b; Edwards et al., 2012, 2014; Marritt et al., 2012a,b; McMillan et al., 2012, 2013). They are key components of the pathways that collectively mediate electron transfer between quinone/quinol pool in the cytoplasmic membrane and the extracellular electron donors or acceptors, whose electron transfer processes are spanning the entire width of bacterial cell envelope (Liu et al., 2012; Richardson et al., 2012; Shi et al., 2012a,b). Similar to the *mtr* gene clusters, additional genes encoding *c*-Cyt, nearly all of which possessed >one heme-binding motifs, often associated with the *pcc* gene clusters (Figure 1). Given the lack of sequence conservation among *Geobacter c*-Cyts as well as among the Pcc *c*-Cyts (Butler et al., 2010; Liu et al., 2014), it is not surprising that these additional *c*-Cyts show no apparent sequence similarity to the *c*-Cyts that are proposed to be involved in quinol oxidation in the cytoplasmic membrane, electron transfer in the periplasm or extracellular reduction of Fe(III) in *Geobacter* spp. (Lovley, 2006, 2012). Lack of sequence conservation among the Pcc *c*-Cyts suggests that different Pcc complexes may interact with different periplasmic and outer-membrane *c*-Cyts for intermolecular electron transfer (Liu et al., 2014). Thus, it is reasonable to hypothesize that some of these Pcc-associated *c*-Cyts are also key components of pathways that transfer electrons between the quinone/quinol pool in the cytoplasmic membrane and substrates external to the bacterial cells.

Consistent with this speculation, the *c*-Cyts encoded by the *pcc*-associated genes are predicted to be localized in the cytoplasmic membrane (Dester_ 0351, Geob_1683, and Theam_0871), the periplasm (IALB_1840 and GSU_2645) and the outer membrane (GM18_3465) (Figure 1), similar to the *c*-Cyts encoded by the *mtr*-associated genes (Shi et al., 2012b). Notably, the predicted cytoplasmic membrane *c*-Cyts Dester_0351 of *D. thermolithotrophum* DSM 11699 and Theam_0871 of *T. ammonificans* HB-1are 79% identical and each protein contains six typical heme-binding motifs (CX_2_CH), two atypical heme-binding motif (CX_3−5_CH) of the *c*-Cyt and 24 histidine residues in which 16 are the putative ligands for *c*-type hemes. Furthermore, BLAST search identifies low sequence similarity between Dester_0351/Theam_0871 and cytochrome *b* subunits of bacterial formate dehydrogenases that also use histidine residues as heme ligands (Gross et al., 2004). Thus, some of the extra histidine residues found in the amino acid sequences of Dester_0351 and Theam_0871 may be the ligands for the *b*-type hemes. The cytochrome *b* subunits of bacterial formate dehydrogenase are the cytoplasmic membrane proteins with quinone reduction activity in which the hemes are involved (Gross et al., 2004). Previously, we found that the genes encoding the *c*-Cyts with sequence similarity to cytochrome *b* subunits of bacterial formate dehydrogenase (MtrH/MtoC) are part of the *mtr* gene clusters where they are proposed to be involved in quinone/quinol cycling in the cytoplasmic membrane (Shi et al., 2012b). Although Dester_0351/Theam_0871 and MtrH/MtoC share very low sequence identity (<17%) in which most identity is in the regions of their heme-binding motifs, Dester_0351 and Theam_0871 may also be involved in quinone/quinol cycling in the cytoplasmic membrane, similar to MtrH and MtoC.

**Figure 4:**
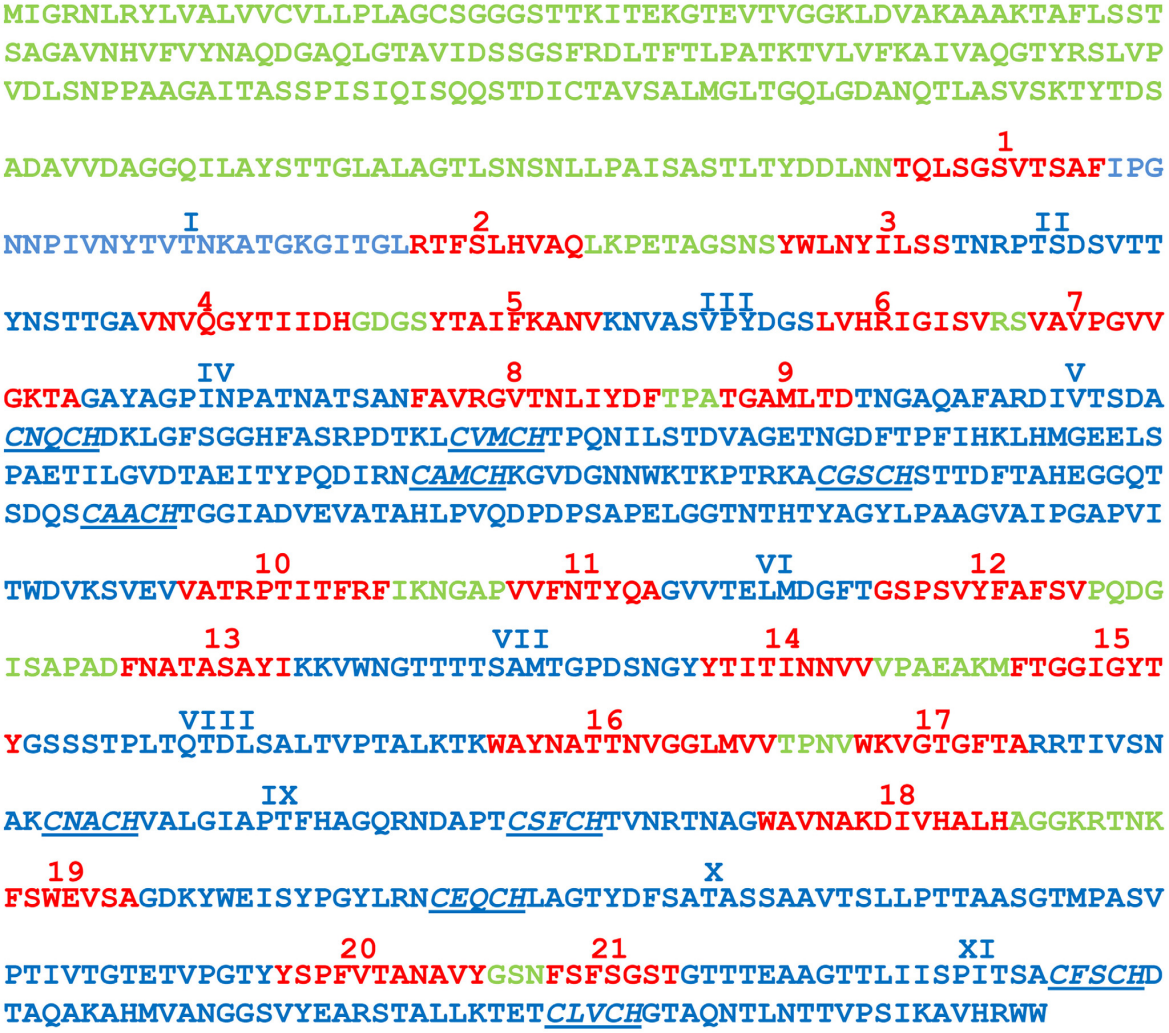
**The amino acid sequence of GM18_3465 of *Geobacter* sp. M18**. The N-terminus and predicted short solvent-exposed loops are in green, the predicted trans-outer membrane motifs are in red, and predicted long solvent-exposed loops are in blue. The 21 trans-outer membrane motifs are numbered sequentially and the numbers are displayed in red and Arabic numerals and above the amino acid sequence. The long solvent-exposed loops are numbered sequentially and numbers are displayed in blue and Roman numerals and above the amino acid sequence. The heme-binding motifs are underlined and in italic.

Interestingly, GM18_3465 of *Geobacter* sp. M18 is predicted to be a porin-like, 10-heme and outer-membrane *c*-Cyt with 21 trans-outer membrane motifs by the Hidden Markov Model with the posterior decoding method using a dynamic programming algorithm. The posterior decoding method using a dynamic programming algorithm is better in prediction than that of Viterbi and N-best algorithms (Bagos et al., 2004a,b), which also predict that GM18_3465 is a porin-like outer-membrane protein with different trans-outer membrane motifs. All the heme-binding motifs are found in the long solvent-exposed loops: five heme-binding motifs in loop 5, two each in loop 9 and 11 and one in loop 10 (Figure 4). Although the porin-cytochrome is a common mechanism shared by different groups of Gram-negative bacteria for transferring electrons across the outer membrane, all previously identified and characterized porin-cytochrome proteins are complexes that each consists or is predicted to consist of a porin-like outer-membrane protein, a periplasmic *c*-Cyt and in most cases an outer-membrane *c*-Cyt (Hartshorne et al., 2009; Liu et al., 2012, 2014; Richardson et al., 2012; Shi et al., 2012b; White et al., 2013). The current porin-cytochrome model proposes that the porin-like outer-membrane proteins function as scaffolds through which the *c*-Cyts are inserted (Richardson et al., 2012; Liu et al., 2014). To the best of our knowledge, GM18_3465 is the first reported case of a putative porin-like outer-membrane *c*-Cyt with multiple hemes. Based on the current porin-cytochrome model, we propose that GM18_3465 may contain a cytochrome domain and a trans-outer membrane domain into which the cytochrome domain may also be inserted for mediating trans-outer membrane electron transfer. A key question is whether GM18_3465 alone can transfer electrons across the outer membrane.

Other genes associated with the identified *pcc* gene clusters include those encoding hypothetical proteins and a putative chitinase, in addition to the genes encoding the transcription factors OrfR and OrfS in the *omcB*-associated gene clusters of *G. sulfurreducens* PCA, where OrfR regulates expression of *ombB-omaB-omcB* gene cluster (Leang and Lovley, 2005) and OrfS may regulate expression of *ombC-omaC-omcC* gene cluster (Figure 1).

### The pcc porin-like outer-membrane proteins and periplasmic *c*-Cyts

In the Mtr system, all identified porin-like outer-membrane proteins (i.e., MtrB/MtoB) contain 28 predicted trans-outer membrane motifs, including PioB of the phototrophic Fe(II)-oxidizing bacterium *Rhodopseudomonas palustris* TIE-1. All identified periplasmic *c*-Cyts (i.e., MtrA/MtoA/PioA) possess or are predicted to possess 10 hemes (Pitts et al., 2003; Shi et al., 2005, 2012b; Jiao and Newman, 2007; Fredrickson et al., 2008; Hartshorne et al., 2009; Liu et al., 2012; White et al., 2013). In the Pcc system, predicted trans-outer membrane motifs found in the porin-like outer-membrane proteins varied, ranging from 18 to 22, while predicted heme-binding motifs in the periplasmic *c*-Cyts also varied, ranging from 5 to 14 (Figures 2A,B) (Liu et al., 2014). Consequently, the Pcc porin-like outer-membrane proteins are much smaller than MtrB/MtoB/PioB and are predicted to form the pores on the outer membrane that may also be smaller than those formed by MtrB/MtoB/PioB. The amino acid sequence identity among Pcc porin-like outer-membrane proteins and periplasmic *c*-Cyts also vary greatly, ranging from 8 to100% and from 13 to100%, respectively (Tables S1–S4). Despite the sequence differences, phylogenetic analyses revealed that both Pcc porin-like outer-membrane proteins and periplasmic *c*-Cyts were clustered into five different groups, except IALB_1839 and IALB_1838 of *I. album* JCM 16511 and Kuster_4034 and Kuster_4025 of *Candidatus* Kuenenia stuttgartiensis, which are distantly related to the rest of their respective counterparts (Figures 2A,B). Within each phylogenetic group, the proteins are often more closely related to each other than to those in the different groups (Figures 2A,B and Tables S1–S4). Remarkably, the porin-like outer-membrane protein and periplasmic *c*-Cyt from the same gene cluster are always found in similar corresponding phylogenetic groups. For instance, OmbB and OmbC of *G. sulfurreducens* PCA are in Group I of the porin-like outer-membrane proteins, while OmaB and OmaC of *G. sulfurreducens* PCA are in Group I of the periplasmic *c*-Cyts (Figures 1, 2A,B). These results suggest that within their respective phylogenetic groups, the Pcc porin-like outer-membrane proteins and periplasmic *c*-Cyts may be co-evolved.

The Pcc porin-like outer-membrane proteins and periplasmic *c*-Cyts from *Geobacter* spp. are found in their respective phylogenetic Group I and V. The Pcc porin-like outer-membrane proteins and periplasmic *c*-Cyts of *Desulfurivibrio alkaliphilus* AHT2, *Desulfuromonas acetoxidans* DSM 684, and *Desulfurispirillum indicum* S5 are placed in their respective phylogenetic Group II, while those of *Denitrovibrio acetiphilus* DSM 12809, *Desulfurobacterium thermolithotrophum* DSM 11699, and *Thermovibrio ammonificans* HB-1 are in the Group III. Phylogenetic Group IVs include Pcc porin-like outer-membrane proteins and periplasmic *c*-Cyts of the *Anaeromyxobacter* spp. analyzed (Figure 2).

As discussed in the Overview section, the *pcc* gene clusters of *A. dehalogenans* 2CP-1, *A. dehalogenans* 2CP-C, and *Anaeromyxobacter* sp. K and *Candidatus* Kuenenia stuttgartiensis lack the genes encoding the outer-membrane *c*-Cyts. Lack of the outer-membrane *c*-Cyts maybe one of the reasons that the Pcc periplasmic *c*-Cyts associated with these bacteria are larger and have more heme-binding motifs than rest of the periplasmic *c*-Cyts. An exception is IALB_1838 of *I. album* JCM 16511 that is the largest Pcc periplasmic *c*-Cyt identified to date, which possesses 400 amino acid residues and 14 heme-binding motifs (Figure 2B). With extra hemes, these larger periplasmic *c*-Cyts could transfer electrons across the outer-membrane in the absence of outer-membrane *c*-Cyts.

### The pcc outer-membrane *c*-Cyts

In the Mtr system, all the outer-outer membrane *c*-Cyts (i.e., MtrC) have or are predicted to have10 hemes (Shi et al., 2006, 2012b; Hartshorne et al., 2007; Fredrickson et al., 2008; Clarke et al., 2011). In the Pcc system, the typical heme-binding motifs (i.e., CX_2_CH) found in the outer-membrane *c*-Cyts varied from 1 to 15 (Figure 3) (Liu et al., 2014). We noticed that each of GM18_3461 and GM18_3467 of *Geobacter* sp. M18 contained only one typical heme-binding motif, while each of their corresponding periplasmic *c*-Cyts had five typical heme-binding motifs (Figures 2B, 3). The combined 6 typical hemes associated with these proposed protein complexes would not form the heme-based electron conduits that are sufficiently long to span entire width of a typical Gram-negative bacterial outer membrane. Further analyses revealed that in addition to a typical heme-binding motif, each of GM18_3461 and GM18_3467 contained five atypical binding motifs with sequences of CX_3−4_CH, which were previously confirmed to bind heme covalently (Stevens et al., 2004). Thus, these *c*-Cyts may bind up to 6 hemes covalently. Given that the 10-heme MtrA/MtoA *c*-Cyt alone could transfer electrons across the outer membrane and the lipid-bilayer of proteoliposomes (Hartshorne et al., 2009; Liu et al., 2012; White et al., 2013), the Pcc protein complex of *Geobacter* sp. M18 that is predicted to consist of a 5-heme periplasmic *c*-Cyt and a 6-heme outer-membrane *c*-Cyt should have enough hemes to form the conduits for the efficient transfer of electrons across the outer membrane.

In addition to GM18_3461 and GM18_3467 of *Geobacter* sp. M18, atypical heme-binding motifs (i.e., CX_3−15_CH) are also found in the Pcc outer-membrane *c*-Cyts GSU_2724 of *G*. *sulfurreducens* PCA, Gura_1837 of *G. uraniireducens* Rf4, Geob_1686 of *Geobacter* sp. FRC-32, and IALB_1837 of *I. album* JCM 16511 (Figure 3). To date, no atypical heme-binding motif has been found in the Pcc periplasmic *c*-Cyts or the Mtr *c*-Cyts. It should be noted that atypical heme-binding motifs are also found in other outer-membrane *c*-Cyts, such as OmcZ of *G. sulfurreducens* PCA (Inoue et al., 2010). It remains to be determined whether the atypical heme-binding motifs with the sequence of CX_>4_CH can also covalently bind hemes.

The identity among the Pcc outer-membrane *c*-Cyts varies from 4 to 100% (Tables S5, S6). The Pcc outer membrane *c*-Cyts are not, however, clustered into distinct phylogenetic groups corresponding to those found in the Pcc porin-like outer-membrane proteins and periplasmic *c*-Cyts (Figures 2, 3). The lack of phylogenetic groups similar to those found in other Pcc components are attributed to the extreme sequence diversity among the Pcc outer-membrane *c*-Cyts. This demonstrates that the Pcc porin-like outer-membrane proteins/periplasmic *c*-Cyts and outer-membrane *c*-Cyts are unlikely co-evolved, which is in contrast to the apparent co-evolution of the Mtr porin-like outer-membrane proteins, periplasmic *c*-Cyts and outer-membrane *c*-Cyts (Shi et al., 2012b). In the Mtr system, without the outer-membrane *c*-Cyt, the porin-like outer-membrane protein and periplasmic *c*-Cyt can work as a single functional unit for mediating electron transfer across the outer membrane (Hartshorne et al., 2009; Liu et al., 2012; Shi et al., 2012b; White et al., 2013). Consistent with these previous findings in the Mtr system are the observations of apparent co-evolution only between Pcc porin-like outer-membrane proteins and periplasmic *c*-Cyts within their phylogenetic groups and the *pcc* gene clusters encoding only Pcc porin-like outer-membrane proteins and periplasmic *c*-Cyts in this study.

### Biological implications

In addition to *Geobacter* spp., other Fe(III)-reducing bacteria identified with the Pcc proteins included *A. dehalogenans* 2CP-1, *A. dehalogenans* 2CP-C, and *Anaeromyxobacter* sp. K and *D. acetoxidans* DSM 684. Notably, the abundance of the Pcc periplasmic *c*-Cyt Adeh_3392 of *A. dehalogenans* 2CP-C increased under Mn(IV)-reducing conditions, compared to that when Fe(III)-citrate was provided as a terminal electron acceptor (Nissen et al., 2012), which is consistent with the proposed role of Adeh_3392 in extracellular electron transfer by *A. dehalogenans* 2CP-C. Identification of the *pcc* gene cluster in *D. acetoxidans* DSM 684 is also consistent with previous findings that *D. acetoxidans* DSM 684 was phylogenetically related to *G. metallireducens* and *c*-Cyts were involved in reduction of solid-phase Fe(III) or Mn(IV) oxides by *D. acetoxidans* DSM 684 (Roden and Lovley, 1993).

Among other bacteria with *pcc* gene clusters, only *D. alkaliphilus* AHT2 and *I. album* have been tested for their ability to grow on Fe(III) or Mn(IV) oxides and were found to be unable to use either as a terminal electron acceptor (Sorokin et al., 2008; Iino et al., 2010). It remains unknown whether the remaining bacteria with *pcc* gene clusters can use Fe(III) and Mn(III, IV) oxides as the terminal electron acceptors. However, it should be pointed out that *pcc* gene clusters may not be restricted to mediation of extracellular reduction of Fe(III) and Mn(III, IV) oxides. Mtr proteins are directly involved in extracellular reduction of dimethyl sulfoxide and extracellular oxidation of Fe(II), in addition to extracellular reduction of Fe(III) and Mn(III, IV) oxides (Gralnick et al., 2006; Jiao and Newman, 2007; Liu et al., 2012, 2013; Shi et al., 2012b). Similarly, the Pcc proteins found in the bacteria that are not known to reduce Fe(III) or Mn(III, IV) oxides may also be involved in extracellular electron transfer reactions with other substrates. A common trait shared by *D. alkaliphilus* AHT2, *D. thermolithotrophum* DSM 11699, and *T. ammonificans* HB-1 is their utilization of insoluble S^0^ as the terminal electron acceptor (L'Haridon et al., 1998; Sorokin et al., 2008; Giovannelli et al., 2012). The ability of these bacteria to reduce S^0^ extracellularly may be attributed in part to their possession of the Pcc proteins.

The SeO^2−^_4_- and SeO^2−^_3_-respiring bacterium *D. indicum* S5 reduces water soluble SeO^2−^_4_ and SeO^2−^_3_ to water insoluble selenium (Se^0^), which forms Se^0^-containing granules outside the bacterial cells (Narasingarao and Haggblom, 2007; Rauschenbach et al., 2011a). Although they were once thought to be localized in the periplasm, the terminal reductases for SeO^2−^_4_ and SeO^2−^_3_ in *D. indicum* S5 have not been identified (Rauschenbach et al., 2011b). Formation of Se^0^-containing granules outside the bacterial cells after reduction of SeO^2−^_4_ and SeO^2−^_3_ by *D. indicum* S5 and existence of the *pcc* gene cluster in *D. indicum* S5 collectively suggest that the reduction of SeO^2−^_4_ and SeO^2−^_3_ may occur extracellularly. Hence, we suggest that Pcc proteins Selin_2480, Selin_2481, and Selin_2482 are associated with the outer membrane where they catalyze extracellular reduction of SeO^2−^_4_ and SeO^2−^_3_. Extracellular reduction of SeO^2−^_4_ and SeO^2−^_3_ will avoid accumulation of insoluble Se^0^ intracellularly, which may be detrimental to the cells of *D. indicum* S5. This is very similar to microbial extracellular reduction of chromium and uranium, which is considered as a detoxification mechanism (Belchik et al., 2011; Cologgi et al., 2011).

It should be pointed out that unlike the *mtr* gene clusters that are also found in genomes of the Fe(II)-oxidizing bacteria (Jiao and Newman, 2007; Liu et al., 2012, 2013; Shi et al., 2012b; Emerson et al., 2013), to date no *pcc* gene cluster has been identified in any known Fe(II)-oxidizing bacterium.

## Conclusions

In addition to their shared similarities in biological functions and protein compositions, the Pcc and Mtr systems also share similar traits in their genetic organizations, such as association with additional genes encoding *c*-Cyts. Analyses of the amino acid sequences of the Pcc-associated *c*-Cyts suggest that they may be involved in redox cycling of quinone/quinol pool in the cytoplasmic membrane and electron transfer across the periplasm and outer membrane. The first two proposed functions of the Pcc-associated *c*-Cyts are very similar to the proposed functions of some Mtr-associated *c*-Cyts. Together, these shared similarities suggest that *c*-Cyts play critical roles in mediation of electron transfer across not only the outer membrane but also the periplasm as well as redox cycling of quinone/quinol pool in the cytoplasmic membrane in both Pcc- and Mtr-mediated electron transfer pathways. Although it still lacks experimental verification, genomic analyses of the *pcc* gene clusters suggest that the Pcc system may be involved in extracellular electron transfer reactions with the substrates other than Fe(III) and Mn(III, IV) oxides, such as S^0^, SeO^2−^_4_, and SeO^2−^_3_, which is also very similar to the Mtr system that is involved in extracellular reduction of dimethyl sulfoxide and extracellular oxidation of Fe(II) in addition to extracellular reduction of Fe(III) and Mn(III, IV) oxides.

Major differences are found between the Pcc and Mtr systems. In the Mtr system, apparent co-evolution among the porin-like outer-membrane proteins, periplasmic *c*-Cyts and the outer-membrane *c*-Cyts is suggested (Shi et al., 2012b). In the Pcc system, extremely diverse amino acid sequences of the outer-membrane *c*-Cyts indicate no apparent co-evolution between the outer-membrane *c*-Cyts and the porin-like outer-membrane proteins/the periplasmic *c*-Cyts. This observation supports pervious suggestions that the Pcc and Mtr systems evolve independently (Liu et al., 2014). Frequent detections of the atypical heme-binding motifs in the Pcc outer-membrane *c*-Cyts is another unique feature of the Pcc system. Compared to those with the typical heme-motifs, the outer-membrane *c*-Cyts with atypical heme-binding motifs are much less characterized. Thus, future research should focus on the detailed characterizations of biochemical, biophysical and electrochemical properties of these *c*-Cyts with atypical heme-binding motifs.

### Conflict of interest statement

The authors declare that the research was conducted in the absence of any commercial or financial relationships that could be construed as a potential conflict of interest.
